# Lenalidomide and Dexamethasone in Scleromyxedema: A Case Report of Long-Term Efficacy and Challenges in a 54-Year-Old Patient

**DOI:** 10.7759/cureus.63181

**Published:** 2024-06-26

**Authors:** Supriya Peshin, Shivani K Modi, Nicholas Burwick

**Affiliations:** 1 Internal Medicine, Norton Community Hospital, Norton, USA; 2 Internal Medicine, Einstein Medical Center Philadelphia, Norristown, USA; 3 Hematology, University of Washington, Seattle, USA

**Keywords:** mgus, dexamethasone, lichen myxedematous, lenalidomide, scleromyxedema

## Abstract

Lichen myxedematosus (LM) is characterized by dermal mucin buildup, fibroblast proliferation, and variable presentation. The diffuse variant, known as scleromyxedema, is identified by monoclonal paraprotein presence and lack of thyroid issues, with considerations for infections and underlying conditions. Despite lacking FDA-approved treatment, intravenous immunoglobulin stands as effective, although resource-intensive, while targeting the clonal source of pathogenic immunoglobulin offers an alternate therapeutic route. Our case explores the efficacy of an oral plasma cell-focused lenalidomide regimen, inducing relief and treatment-free periods, while still facing relapses. However, this approach poses risks, necessitating a comparative safety and efficacy assessment. Data remains insufficient to establish the prolonged efficacy of plasma cell-targeted therapy versus alternatives for monoclonal gammopathy of undetermined significance-associated scleromyxedema. We present the case of a 54-year-old male patient diagnosed with LM managed for six years with relapsing and remitting symptoms.

## Introduction

Lichen myxedematosus (LM) is a rare skin disorder characterized by mucin buildup, fibroblast proliferation, and variable presentations. It accounts for less than 1% of all mucinoses, which themselves are uncommon skin disorders characterized by abnormal deposition of mucin in the skin. The exact prevalence of scleromyxedema is not well defined due to its rarity, but it is estimated to affect fewer than 1 in 1,000,000 individuals worldwide. This condition primarily affects adults in their fifth and sixth decades of life and presents with widespread, firm papules and nodules that may merge into plaques. Typically, these lesions appear on the head, neck, trunk, and extremities, often arranged in linear patterns. The skin surrounding these lesions may exhibit a sclerodermoid appearance, adding to the diagnostic challenge. Despite its primarily cutaneous manifestations, scleromyxedema can extend beyond the skin, involving multiple organ systems. This systemic involvement, observed in a significant percentage of patients, includes neurological manifestations like encephalopathy, seizures, and peripheral neuropathy, potentially related to paraproteinemia. Additionally, scleromyxedema can affect the cardiovascular, pulmonary, gastrointestinal, and musculoskeletal systems, with presentations ranging from myocardial ischemia to esophageal dysmotility [1].

Diagnosing scleromyxedema involves histopathological examination revealing mucin deposition, fibroblast proliferation, and fibrosis, along with evidence of monoclonal gammopathy, typically IgG λ. The disease course is chronic, often with a poor response to therapy, necessitating empirical treatment approaches such as intravenous immunoglobulin (IVIG), plasmapheresis, thalidomide, and corticosteroids. Given its rarity and diverse clinical presentations, scleromyxedema poses challenges in both diagnosis and management. Understanding its complex pathogenesis, which involves cytokine-mediated stimulation of fibroblasts and glycosaminoglycan synthesis, remains crucial for advancing therapeutic strategies and improving patient outcomes [1].

The diffuse variant, scleromyxedema, involves monoclonal paraproteins and no thyroid abnormalities, with considerations for infections and underlying conditions [2]. Despite the lack of FDA-approved treatments, IVIG is effective but resource intensive. An alternative approach targets the clonal source of pathogenic immunoglobulins [1]. Other alternatives include systemic corticosteroids like prednisone or dexamethasone to mitigate inflammation and immune responses, although long-term use is constrained by potential adverse effects. Thalidomide, known for its anti-inflammatory properties, is employed cautiously due to the risks of teratogenicity and neuropathy. In refractory cases, melphalan, an alkylating agent, may be considered to suppress abnormal cell growth, despite the associated risks of myelosuppression and secondary malignancies. Plasmapheresis, aimed at eliminating circulating paraproteins and serum factors implicated in disease pathogenesis, is frequently combined with IVIG for enhanced efficacy. Topical treatments, such as steroids or emollients, offer localized symptom relief but do not target systemic involvement. Immunomodulatory agents like lenalidomide (Ld) and rituximab show varying efficacy in modulating immune responses and have been explored in limited studies. Supportive care plays a pivotal role in managing complications such as neuropathy, cardiac issues, and gastrointestinal symptoms, emphasizing personalized approaches to enhance the quality of life amidst the challenges posed by scleromyxedema [1]. This case report explores the efficacy of an oral Ld regimen in a 54-year-old male patient with scleromyxedema, showing significant symptom relief and treatment-free periods yet facing relapses. This highlights the need for further comparative safety and efficacy assessments.

## Case presentation

We present the case of a 54-year-old male with a history of Grave’s disease, although his thyroid-stimulating hormone levels had normalized post-thyroid ablation and replacement therapy. He presented with a two-month history of erythematous pruritic eruptions involving the face, upper extremities, and torso. The rash had started one week after the initiation of allopurinol for gout. A biopsy of the lesional skin was performed and revealed superficial dermal spindle cell proliferation associated with thickened collagen bundles and interstitial mucin deposits. A diagnosis of interstitial granulomatous drug reaction was favored. All medications, including allopurinol, were discontinued. The patient was started on topical triamcinolone ointment and a prednisone taper, which resulted in the subsequent improvement of symptoms. However, after completing the taper and reinitiating antihypertensives due to uncontrolled hypertension, he experienced a recurrence of small pruritic papules, erythema, and edema of the face and shoulders. On presentation to the clinic, the patient reported a three-month history of skin tightening and hardening of his forehead, neck, back, torso, and arms. He had a decreased ability to open his mouth due to skin stiffness, intermittent abdominal swelling, mild shortness of breath, and hand and finger swelling with difficulties making a fist. The medications he was taking at the time of presentation included atenolol, levothyroxine, hydrochlorothiazide, simvastatin, and losartan.

Physical examination revealed exaggerated wrinkling of the glabella and firm skin on the sides of the nose, upper back, and shoulders. Numerous 2-3 mm waxy papules were diffusely scattered over the upper shoulders. There was notable tightness along the labial commissures, with the ability to insert 2.5 fingers between his incisors at maximal opening. His skin appeared xerotic and had become notably flushed with minimal movement, demarcating areas of induration. Periorbital edema, telangiectasia, and calcinosis were absent.

A cutaneous punch biopsy specimen from the right shoulder was performed. The most prominent abnormality was a bandlike zone of stromal hypercellularity and thickened collagen bundles in the upper reticular dermis. Histiocytoid cells with abundant blue-gray cytoplasm and spindle cells surrounded thickened collagen bundles, occasionally forming loose granulomas and cords, without necrobiotic foci. There was also a mild perivascular lymphoid infiltrate with rare plasma cells. Colloidal iron statins highlighted increased dermal mucin, which cleared with hyaluronidase digestion. Immunohistochemical staining for CD68 highlighted the lesional histiocytoid and spindled cells. The epidermis showed minimal alterations, including mild compact hyperkeratosis and attenuation of the rete ridges. These histopathologic features were suggestive of interstitial granuloma annulare.

Diagnostic assessment revealed monoclonal paraproteinemia (0.2 g/dL IgG lambda and 0.1 g/dL IgA lambda), accompanied by normal serum-free light chains, and lacking multiple myeloma diagnostic criteria. Treatment commenced with Ld and dexamethasone [2], known for their efficacy in plasma cell-directed therapy. Herein, we present data spanning six years of disease management, culminating tragically in his demise due to metastatic melanoma.

**Figure 1:**
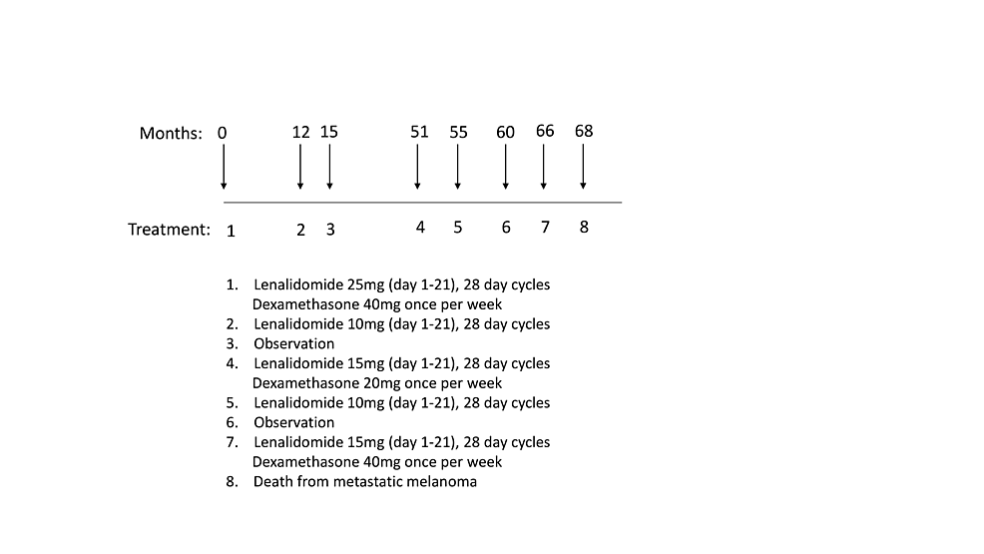
Treatment timeline The course of treatment is outlined from its initiation (zero months) to the date of death at 68 months. The detailed treatment course is provided below the timeline.

Following treatment initiation, the patient experienced swift symptomatic relief. He underwent 12 months of combination therapy with Ld, followed by a three-month maintenance phase of Ld monotherapy, sustaining therapeutic benefits. Notably, the IgG lambda monoclonal protein remained detectable solely via immunofixation. However, an episode of bacterial pneumonia necessitated hospitalization, marked by an elevated white blood cell count of 14.1 k/μL and a maximum temperature of 100.9 °F, prompting discontinuation of Ld in favor of an observational approach. Three years elapsed before scleromyxedema recurrence occurred, with a monoclonal protein level of 0.13 g/dL of IgG lambda. Resumption of Ld therapy at reduced doses (Figure 1) elicited rapid disease improvement. Despite transitioning to observation after nine months of therapy, scleromyxedema recurred within six months. Tragically, although the patient responded promptly to Ld reinitiation, progressive headaches emerged, leading to the discovery of widespread metastatic melanoma and ultimately leading to his demise.

## Discussion

LM is a condition marked by the accumulation of mucin in the skin layers, along with fibroblast growth and tissue scarring. This ailment can manifest in localized, diffuse, or atypical forms [1]. The diffuse variant, recognized as scleromyxedema, is characterized by the existence of monoclonal paraproteins and the absence of thyroid irregularities. Potential factors such as HIV, HCV, autoimmune diseases, and hidden malignancies should be taken into consideration [2,3]. Regrettably, there is no FDA-approved remedy for scleromyxedema, contributing to the generally unfavorable prognosis for afflicted patients. The utilization of IVIG has demonstrated efficacy as a treatment, but its availability is limited. Moreover, continuous clinic visits for IV infusions are necessary, and relapses are common upon cessation of treatment [4].

Another core therapeutic avenue revolves around addressing the clonal lesion believed to be responsible for pathological immunoglobulin production. This strategy encompasses various treatments such as chemotherapy, immunomodulatory agents, corticosteroids, and other targeted therapies [3,5].

In our case, employing an oral regimen targeting plasma cells, specifically Ld and dexamethasone, exhibited swift alleviation of scleromyxedema symptoms and enabled a prolonged treatment-free period. However, it was observed that scleromyxedema consistently resurged upon discontinuation of treatment. While Ld and dexamethasone prove effective against plasma cell dyscrasias, this regimen does carry an elevated risk of secondary cancers and susceptibility to infections [6]. Further research is warranted to ascertain plasma cell-directed therapy’s enduring safety and efficacy compared to IVIG or other strategies for managing monoclonal gammopathy of undetermined significance (MGUS)-associated scleromyxedema.

## Conclusions

LM, particularly its diffuse variant, scleromyxedema, poses significant therapeutic challenges due to the lack of FDA-approved treatments. While IVIG has shown effectiveness, its resource demands limit its use. Our case demonstrates that an oral regimen of Ld and dexamethasone can provide symptom relief and extended treatment-free periods, although relapses are common upon cessation. This treatment approach also carries the risk of secondary cancers and infections. Consequently, further research is essential to compare the long-term safety and efficacy of plasma cell-targeted therapy with IVIG and other treatment strategies for MGUS-associated scleromyxedema.
